# Atypical Triplane Fracture—Anteromedial Epiphyseal Sleeve Avulsion Pattern

**DOI:** 10.7759/cureus.17934

**Published:** 2021-09-13

**Authors:** Jean H Nel, Conrad F Nel

**Affiliations:** 1 Radiology, University of Cambridge School Of Clinical Medicine, Cambridge, GBR; 2 Radiology, Mid Yorkshire Hospitals NHS Trust, Wakefield, GBR

**Keywords:** atypical triplane fracture, anteromedial epiphyseal sleeve avulsion, extra-articular, medial malleolus, salter-harris

## Abstract

Atypical triplane fractures are unusual, but when they occur, the anteromedial epiphyseal sleeve avulsion pattern appears to be the most frequent. We present a classic case of the latter, which supports the introduction of an additional category to the classification of these fractures.

## Introduction

Triplane fractures are relatively uncommon, representing only 6% of paediatric ankle fractures [1]. As the name indicates, they classically extend through three planes of the distal tibia: coronally or oblique coronally through the metaphysis, axially through the physeal plate, and sagittally through the epiphysis. The typical fracture configuration extends intra-articularly and represents a Salter-Harris IV fracture, but there are variations in the fracture pattern. Atypical patterns are more unusual and include intra-articular fractures, which involve the non-weight bearing area of the tibial plafond and extra-articular fractures of the medial malleolus [2]. Our case is an example of the latter configuration and provides further evidence in support of a change in the classification system as proposed by Yung et al. [3].

## Case presentation

Patient history and presentation

**Figure 1:**
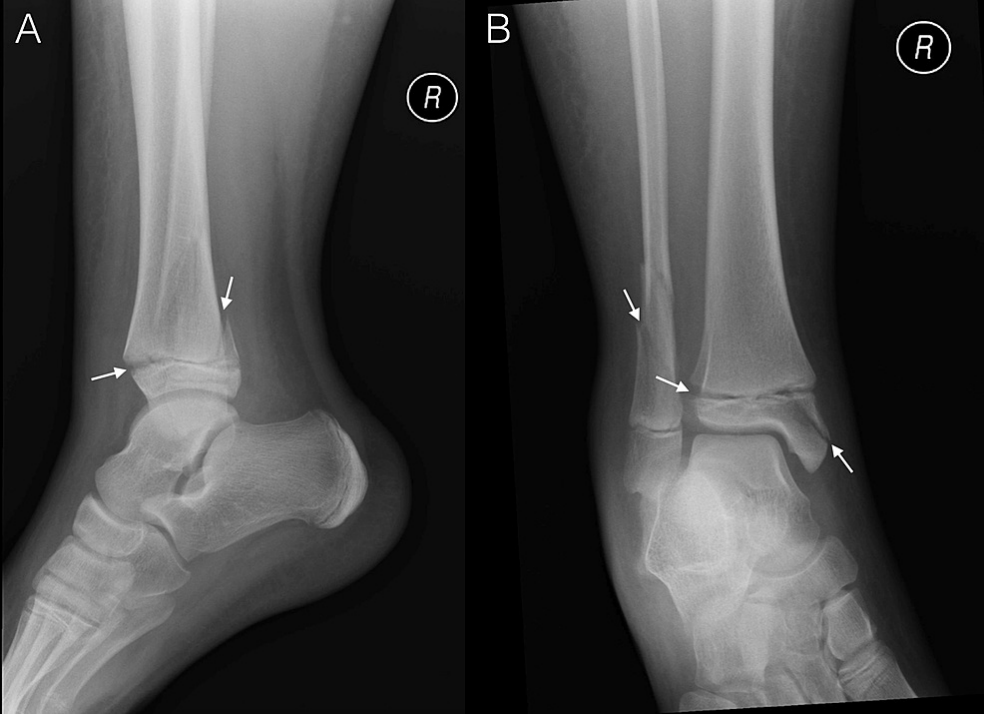
Anteroposterior and lateral x-rays of the right ankle. The radiographs demonstrate a spiral fracture of the distal fibula, mildly displaced fractures of the posterior and medial malleoli, and a degree of physeal widening anterolaterally.

**Figure 2:**
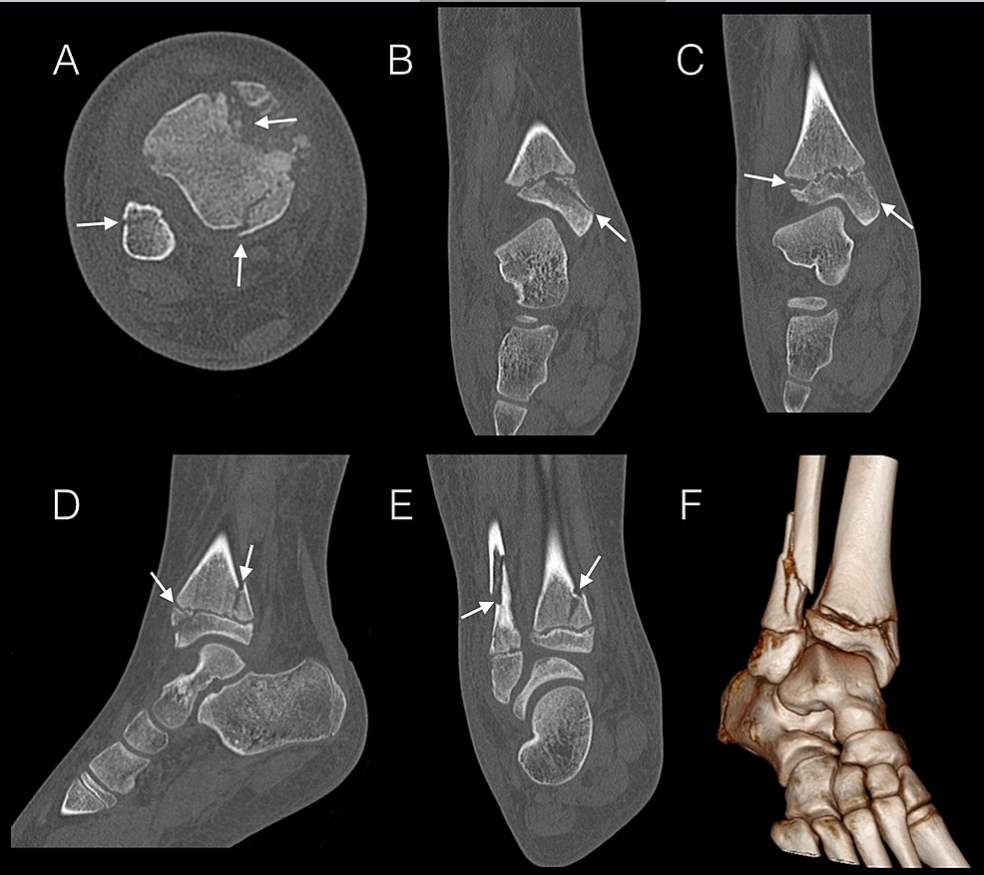
CT of the right ankle. A: Axial reconstruction showing the spiral fracture of the fibula, posterior malleolar fracture, and anteromedial avulsion fracture of the medial malleolus. B: Coronal reconstruction demonstrating the extra-articular anteromedial epiphyseal sleeve avulsion of the medial malleolus. C: Coronal reconstruction demonstrating anterolateral physeal widening and the medial malleolar fracture. D: Sagittal reconstruction demonstrating both the posterior malleolar and extra-articular medial malleolar fractures. E: Coronal reconstruction capturing the posterior malleolar fracture and spiral fracture of the fibula. F: Volume rendered three-dimensional reconstruction.

The patient is a 13-year-old boy who presented to the emergency department (ED) with a history of falling off a scooter four days prior with forced plantar flexion of his foot. Attendance at the ED was delayed as it was thought to be a sprain only; however, he remained non-weight-bearing with increasing bruising and swelling despite icing and elevation. On examination, the skin was intact, there was tenderness of both malleoli, and marked swelling and bruising of the ankle and foot. X-rays demonstrated a spiral fracture of the distal fibula extending to the physeal plate, mildly displaced fractures of the posterior and lateral malleoli, and asymmetry of the physis with anterolateral widening (Figures 1A, 1B). A CT confirmed the radiographically demonstrated fractures with lateral physeal widening and an anteromedial epiphyseal sleeve avulsion fracture of the medial malleolus - findings in keeping with an atypical extra-articular triplane fracture (Figures 2A-2F).

Treatment and course

**Figure 3:**
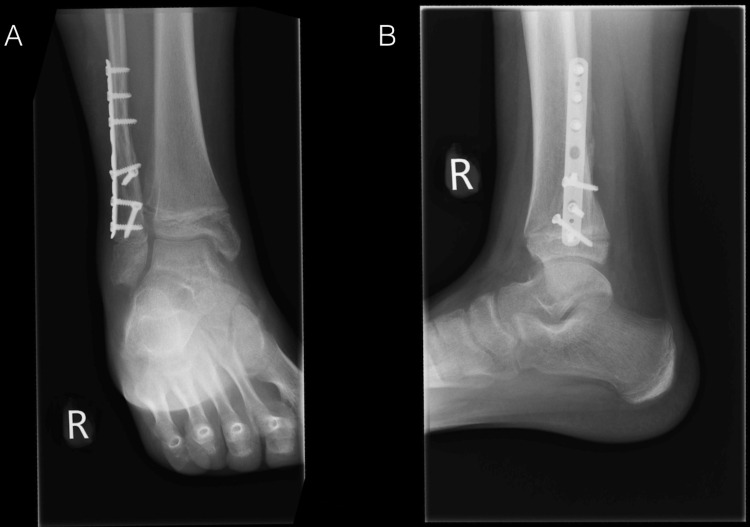
Post-operative anteroposterior and lateral X-rays of the right ankle. Six weeks follow-up. Fibular plate and screws in situ. Good position of the fractures with evidence of healing.

The fibular fracture was treated with open reduction and internal fixation. The syndesmosis was confirmed to be intact and the tibial fracture in a good position, so there was no need for fixation. The patient was rehabilitated non-weight-bearing for six weeks and mobilised in a walker boot. Follow-up X-rays at six weeks demonstrated a good position of the fractures and evidence of fracture healing (Figures 3A, 3B). The patient’s recovery was uneventful and he has resumed normal activity.

## Discussion

Ankle fractures in children are the second most common growth plate fractures in humans and are among the 10 most common reasons for paediatric orthopaedic hospital admissions [4]. Triplane fractures are a relatively small subgroup of paediatric ankle fractures. They are complex fractures involving the closing physis of the distal tibia in adolescents and occur at a younger age in girls than boys, presumably due to earlier physeal closure in girls [5]. The site of first closure of the physis is at a central tibial bump known as Kump’s bump, which overlies the medial edge of the talar hump and the propensity of triplane fractures to extend laterally along the physis is thought to be related to the normal sequence of physeal closure [5]. Multiple different triplane fracture configurations have been described, with variants of two, three, and four fragments [5,6]. Some authors ascribed the different types to different mechanisms of injury; however, von Laer suggested that the degree of maturity of the physeal plate was the determining factor [7]. 

Atypical and extra-articular triplane fractures seem rare, but were probably often overlooked as a variant before and therefore may have been underreported in the literature. We have identified at least two series that showed the medial malleolar variant of triplane fractures to be very common [3,5]. Shin et al. previously described these as intramalleolar fractures and identified three distinct subtypes, which formed the basis of their proposed classification system: (i) intra-articular and within the weight-bearing zone; (ii) intra-articular and outside the weight-bearing zone; and (iii) extra-articular [2]. A more recent paper by Yung et al. revisited this classification and proposed a modified classification of atypical triplane fractures by the addition of a fourth type (the most frequent in their series), which constitutes an anteromedial epiphyseal sleeve (AMES) avulsion fracture of the medial malleolus, but which is different in location to a Tillaux fracture and accompanied by the other elements of a triplane fracture [3]. They proposed the mechanism of injury in these cases to be external rotation or eversion of a plantar-flexed foot, which would fit with our case. Awareness of this variant and its mechanism of injury is important from a management perspective as it generally can be managed conservatively and a decision to operatively manage these fractures is not dictated by articular congruity but based on the degree of displacement. The triplane fracture in our patient was managed conservatively, in accordance with the approach suggested by these authors and had a good clinical outcome [3].

## Conclusions

Although extra-articular triplane fractures are uncommon, the anteromedial epiphyseal sleeve avulsion pattern appears to constitute a significant percentage of these fractures. Our case is an excellent example of the latter and provides further evidence in support of the modified classification system and management protocol proposed by Yung et al. Identifying this variant has important management implications, as unless there is significant displacement, it can be managed conservatively.

## References

[1] Cooperman DR, Spiegel PG, Laros GS (1978). Tibial fractures involving the ankle in children. The so-called triplane epiphyseal fracture. J Bone Joint Surg Am.

[2] Shin AY, Moran ME, Wenger DR (1997). Intramalleolar triplane fractures of the distal tibial epiphysis. J Pediatr Orthop.

[3] Yung CS, Kuong EE, Chow W (2019). A previously unreported type of extra-articular triplane fracture: a revised classification system. J Orthop Surg (Hong Kong).

[4] Crawford AH (2012). Triplane and Tillaux fractures: is a 2 mm residual gap acceptable?. J Pediatr Orthop.

[5] Brown SD, Kasser JR, Zurakowski D, Jaramillo D (2004). Analysis of 51 tibial triplane fractures using CT with multiplanar reconstruction. AJR Am J Roentgenol.

[6] Kim JR, Song KH, Song KJ, Lee HS (2010). Treatment outcomes of triplane and Tillaux fractures of the ankle in adolescence. Clin Orthop Surg.

[7] von Laer L (1985). Classification, diagnosis, and treatment of transitional fractures of the distal part of the tibia. J Bone Joint Surg Am.

